# Reducing endogenous insulin is linked with protection against hepatic steatosis in mice

**DOI:** 10.1038/s41387-020-0114-9

**Published:** 2020-04-14

**Authors:** Md Akheruzzaman, Vijay Hegde, Andrew C. Shin, Nikhil V. Dhurandhar

**Affiliations:** Department of Nutritional Sciences, Texas Tech University, Lubbock, TX USA

**Keywords:** Fat metabolism, Type 2 diabetes

## Abstract

**Background:**

Obesity and type 2 diabetes (T2D) are closely associated with hepatic steatosis (HS), which if untreated can advance to serious liver conditions. Since insulin promotes hepatic lipogenesis, reducing hyperinsulinemia may help in treating HS. E4orf1 is an adenovirus-derived protein that improves glucose clearance independent of insulin, lowers insulin amount required for glucose disposal, and reduces HS. As a next step, we evaluated the mechanism for E4orf1-induced reduction in HS and tested that E4orf1 does not induce hypoglycemia, an important attribute for its application as a potential anti-diabetic agent.

**Methods:**

C57Bl/6J mice that transgenically express E4orf1 in adipose tissue (E4orf-Tg) and wild-type (WT) mice received a chow diet for 6 weeks, followed by a high-fat (HF) diet for additional 10 weeks. Body composition, blood glucose, and serum insulin levels upon glucose load were measured at 0, 6, 7, and 16 weeks. Serum free fatty acid (FFA), triglyceride (TG), and hepatic TG were measured at study termination. We compared histology and the mRNA/protein markers of hepatic and adipose tissue lipid metabolism between the two groups of mice.

**Results:**

On chow diet, both groups remained normoglycemic, but E4orf1 expression reduced insulin response. On HF diet, glycemic control in WT deteriorated, whereas E4orf1 significantly enhanced glycemic control, lowered insulin response, reduced hepatic triglycerides, and serum FFA. Overall, a comparison of hepatic mRNA and/or protein expression suggested that E4orf1 expression significantly decreased de novo lipogenesis (DNL) and intracellular lipid transport and increased fat oxidation and TG export. Adipose tissue mRNA and protein markers suggested that E4orf1 expression lowered DNL and increased lipolysis.

**Conclusion:**

Considering that E4orf1 is not secreted in circulation, we postulate that reduced endogenous insulin in E4orf1 mice indirectly contributes to reduce HS by altering hepatic lipid metabolism, including lipogenesis. This study underscores the possibility of indirectly impacting HS by manipulating adipose tissue metabolism.

## Introduction

Glycemic control, insulin sensitivity/resistance, and hepatic lipid accumulation are intimately linked^1^. Non-alcoholic fatty liver disease (NAFLD) pathogenesis is attributed to multiple factors such as excessive dietary fat intake, insulin resistance, dyslipidemia, central adiposity, gut dysbiosis, and genetic and epigenetic factors, described as the “multiple-hit pathogenesis” theory^2^. Of these factors, either tissue-specific or whole-body insulin resistance is considered to be the key risk factor in NAFLD. During early stages of type 2 diabetes (T2D), insulin resistance results in hyperinsulinemia and hyperglycemia, which increases de novo lipogenesis (DNL) in the liver, and enhances hepatic exposure to free fatty acids (FFAs) and inflammatory adipokines released from the adipose tissue^3^. The risk of NAFLD is twofold higher in individuals with T2D, with an estimated prevalence of 57–80% and increased susceptibility to steatohepatitis and fibrosis^4^. Given the effect of hyperinsulinemia and hyperglycemia on liver fat synthesis, various anti-diabetic drugs alone or in combination have been tested as the treatment options for NAFLD^3,5^. In clinical trials and animal models, drugs such as sodium glucose co-transporter-2 inhibitors, thiazolidinediones, and metformin (with sitagliptin, liraglutide, or insulin) showed mostly modest beneficial effects on ameliorating hepatic steatosis but were more effective when combined with weight loss^5^.

The efficacy of these anti-diabetic drugs against hepatic steatosis might be limited due to their mode of action, which mostly focuses on enhancing glucose clearance and/or insulin production but insulin resistance. Effective management of NAFLD would require improved glycemic control as well as increased insulin sensitivity, which will reduce hyperinsulinemia. Hence, studies are warranted with other potential anti-diabetic therapeutic agents that have distinct mode of action for more effective management of T2D and associated NAFLD.

Here we report the role of Early gene 4 open reading frame-1 (E4orf1), a 125 amino acid protein derived from human adenovirus-36 (Ad36). E4orf1 upregulates the PI3K/AKT/GLUT4 axis in a unique insulin-independent manner, improves glycemic control, and reduces the requirement for endogenous insulin^6^. The discovery of the effect of E4orf1 stems from the initial studies of Ad36. Ad36 infection and its association with human obesity is reported in >14 countries and Ad36 is known to cause adipogenesis in in vitro and in vivo models^7^. Although Ad36 is linked with greater adipogenesis in naturally infected humans and experimentally infected animals, paradoxically, it also provides favorable metabolic features, such as improved glycemic control, better serum lipid profile, and reduced hepatic steatosis^8–12^. Subsequently, the E4orf1 protein of Ad36 was identified to have a similar anti-diabetic effect and reduced hepatic steatosis in high-fat (HF) diet-fed mice^13,14^. In mice, the expression of E4orf1 by transgenic approach or via viral vector reduces hepatic steatosis, improves glycemic control, and reduces the endogenous insulin response to glucose load^13,15,16^. This reduction in insulin is not due to an increase in insulin sensitivity^16,17^ but due to the reduced requirement for insulin^6^.

Mouse models show reduced hepatic steatosis when insulin sensitivity is improved. However, improved insulin sensitivity is also associated with reduced insulin levels. This makes it difficult to determine whether the reduced hepatic steatosis is due to greater insulin sensitivity or lower insulin levels. Our mouse model described above can reduce insulin levels without improving insulin sensitivity^17^ and thus offers a very valuable model to separate the effects of insulin amount from that due to tissue sensitivity to insulin.

We hypothesized that, in the mouse model of E4orf1 expression in adipose tissue, reduced levels of insulin, a lipogenic hormone, will be associated with reduced hepatic lipogenesis and steatosis. Another important aspect of the metabolic effects of E4orf1 is its potential as a drug to treat type 1 or type 2 diabetes^13–16,18,19^. While the effect of E4orf1 on HF-diet induced hyperglycemia is reported^13–15^, it is unclear whether E4orf1 will further reduce glucose levels in normoglycemic mice on a chow diet. The ability to avoid hypoglycemia in a normoglycemic state would be an important attribute for an anti-diabetic agent. Taken together, this study was conducted to determine whether: (a) reduction in insulin levels without altering tissue insulin sensitivity will influence hepatic lipid metabolism, (b) E4orf1 will reduce glucose levels even in normoglycemic mice, and (c) E4orf1 will reduce glucose levels differentially in mice in the presence of a chow diet vs a HF diet.

## Material and methods

### Experimental design and animal model

Considering the strong positive association of age with hepatic steatosis in rodents and humans^20–22^, 30 week-old male C57BL/6J mice that transgenically express Ad36E4orf1 protein (E4orf1-Tg, *n* = 15) in the adipose tissue upon doxycycline induction^13^ and age-matched wild-type (WT, *n* = 15) counterparts (Jackson Laboratory) were offered chow-doxycycline diet (chow-dox, 16% Kcal from fat, dox 600 ppm/kg) for 6 weeks followed by 10 weeks of HF-doxycycline diet (HF-dox, 60% Kcal from fat, dox 600 ppm/kg). All animals were housed in the same room ≤5 mice/cage on a 12‐h light/12‐h dark cycle, had ad libitum access to food, and received humane care. The experimental procedures were approved by Institutional Animal Care and Use Committee at Texas Tech University, Lubbock, TX. Mice were weighed weekly and fat mass was measured using EchoMRI (Echo MRI LLC, Houston, TX) at the baseline, after 6 weeks of chow-dox, 1 week of HF-dox, and 10 weeks of HF-dox diet. At these time points, oral glucose tolerance test (GTT, 2 g/kg body weight) were performed following a 4-h fasting. During GTT, blood glucose levels (mg/dL) were measured by tail bleed at *t* = 0 and following oral glucose load, at *t* = 15, 30, 60, and 120 min using AlphaTrak2 glucose meter, and blood was collected in EDTA-coated microvette (Sarstedt, cat. 16.444.100) for insulin measurement using enzyme-linked immunosorbent assay kit (EMD-Millipore, cat. EZRMI-13K).

At study termination, mice were euthanized using CO_2_ asphyxiation and cervical dislocation. Trunk blood, liver, and adipose tissue depots were collected during necropsy and flash-frozen in liquid nitrogen or stored in RNALater (Invitrogen, cat. no. AM7020) or 10% neutral buffered formalin (NBF) for protein, RNA, or histology analysis.

#### Explanation for the number of mice included

The study was started with 15 E4orf1 Tg mice in the experimental group and 15 WT mice in the control group. One mouse from each group died early in the study during experimental procedure. The experimental group was selected based on the presence of E4orf1 DNA (from tail snip). However, in our experience, the presence of E4orf1 DNA in tail tissue of these mice does not guarantee E4orf1 gene expression in adipose tissue. Hence, to include only those animals that expressed E4orf1 protein, inguinal adipose tissue samples were screened for E4orf1 protein expression upon termination of the experiment. Of the 14 mice initially included in the experimental group based on E4orf1 DNA presence, 6 mice expressed E4orf1 protein in adipose tissue and were considered as the experimental group (Supplementary Fig. 1). WT mice receiving doxycycline were used as the control group.

### FFA and triglyceride (TG) determination

Serum FFAs (mmol/L) were measured using Wako NEFA kits. Serum TG level (mg/L) and hepatic TG content (mg/g) were measured using the Cayman Kit (cat. 10010303).

### Histology

Histological analysis of 10% NBF fixed liver samples from randomly selected three E4orf1-Tg and three WT mice was performed by IDEXX BioAnalytics, Columbia, MO. Liver sections were stained with hematoxylin and eosin (H&E) for examining steatosis and inflammation. Sections were also stained with Picrosirius red stain to visualize fibrosis in the liver samples. Microscopic examination and scoring were performed by an experienced pathologist, blinded to group allocation of mice samples. Observed changes were graded, based on severity, utilizing the grading system for rodent NAFLD^23^. The current study included evaluation of 10 fields to count the number of inflammatory foci instead of 5 fields as indicated previously^23^. The average of 10 fields was used to provide a single inflammation score (0–3) for each mouse. Summary scores were calculated for lesions indicative of NAFLD/nonalcoholic steatohepatitis, including macrovesicular steatosis, microvesicular steatosis, hepatocyte hypertrophy, and inflammation. Inguinal adipose tissue from three E4orf1-Tg and three WT mice was sectioned, two slides for each mouse were stained with H&E, and images were captured using EVOS™ FL Auto Imaging System (cat. AMAFD1000). For each slide, three random images (2048 × 1536 pixel, height × width) were captured, and the Adiposoft program (http://imagej.net/Adiposoft) was used to quantify the number, diameter (μm), and area (μm^2^) of the adipocytes.

### Real-time quantitative PCR

Total RNA was extracted from the liver and adipose tissue using the RNeasy® Plus Universal Mini Kit (cat. 73404). cDNA was synthesized using the Maxima cDNA Synthesis Kit (Thermo Fisher Scientific, cat. K1681) with 1 µg of RNA. The expression level of genes associated with liver and adipose tissue lipid metabolism were determined by quantitative real-time PCR. Specific primers for each gene are listed in Supplementary Table 1. The reverse transcriptase-PCR (RT-PCR) reaction mix had a final volume of 20 µL: 25 ng of cDNA, 450 nM of the forward and reverse primers, and 10 µL of 1× SsoAdvanced™ Universal SYBR® Green Supermix (Bio-Rad Laboratories, cat. 172-5271). PCR reactions were carried out in 96-well plates using the Bio-Rad CFX RT-PCR detection system. All reactions were performed in duplicates. Mouse *B2m* and *Tbp* gene were used as reference for liver and adipose tissue, respectively.

### Western blotting

Protein lysate was extracted from the inguinal adipose tissue depot and liver in modified RIPA buffer. Protein extracts were separated using sodium dodecyl sulfate-polyacrylamide gel electrophoresis gel, transferred on to a nitrocellulose membrane, blocked using 10% non-fat milk in TBST for an hour at room temperature, followed by immunoblotting with primary antibodies for E4orf1, acyl-CoA carboxylase (ACC) (Cell Signaling, 3662S), fatty acid synthase (FASN) (BD Bio, 610963), ATP citrate lyase (ATPCL) (Cell Signaling, 4332S), adipose triglyceride lipase (ATGL) (Cell Signaling, 2138S), Caveolin-1 (BD Biosciences, 610060), Ras (Cell Signaling, 3965S), pAKT (Cell Signaling, 9271L), AKT (Cell Signaling, 4691L), or glyceraldehyde 3-phosphate dehydrogenase (Cell Signaling, 2118S) at 1:1000 dilution. To visualize protein bands, the membrane was treated with Clarity western ECL substrate (Bio-Rad, cat. no. 170–5061) reagent following immunoblotting with appropriate horseradish peroxidase secondary antibody.

### Statistical analysis

The current study could be 80% powered at two sided *α* = 0.05 having *n* = 4 mice in each group. To account for unintended losses during experiments, procedures, and assays, we included 15 mice per group. The power calculation is described in Supplementary Fig. 3. Results are presented as mean ± standard error of the mean. Two groups were compared using Welch’s *t* test assuming unequal variance. Two-way repeated-measures analysis of variance was used to analyze time and treatment effect in GTT and insulin data. Homeostatic model assessment of insulin resistance (HOMA-IR) value was calculated using the equation: HOMA-IR = Fasting blood glucose (mg/dL) × Fasting insulin (ng/mL) × 0.072. The relative amount of all mRNAs was calculated using the 2^−ΔΔCT^ method.

## Results

### Weight and body composition changes following chow and HF feeding

**Fig. 1:**
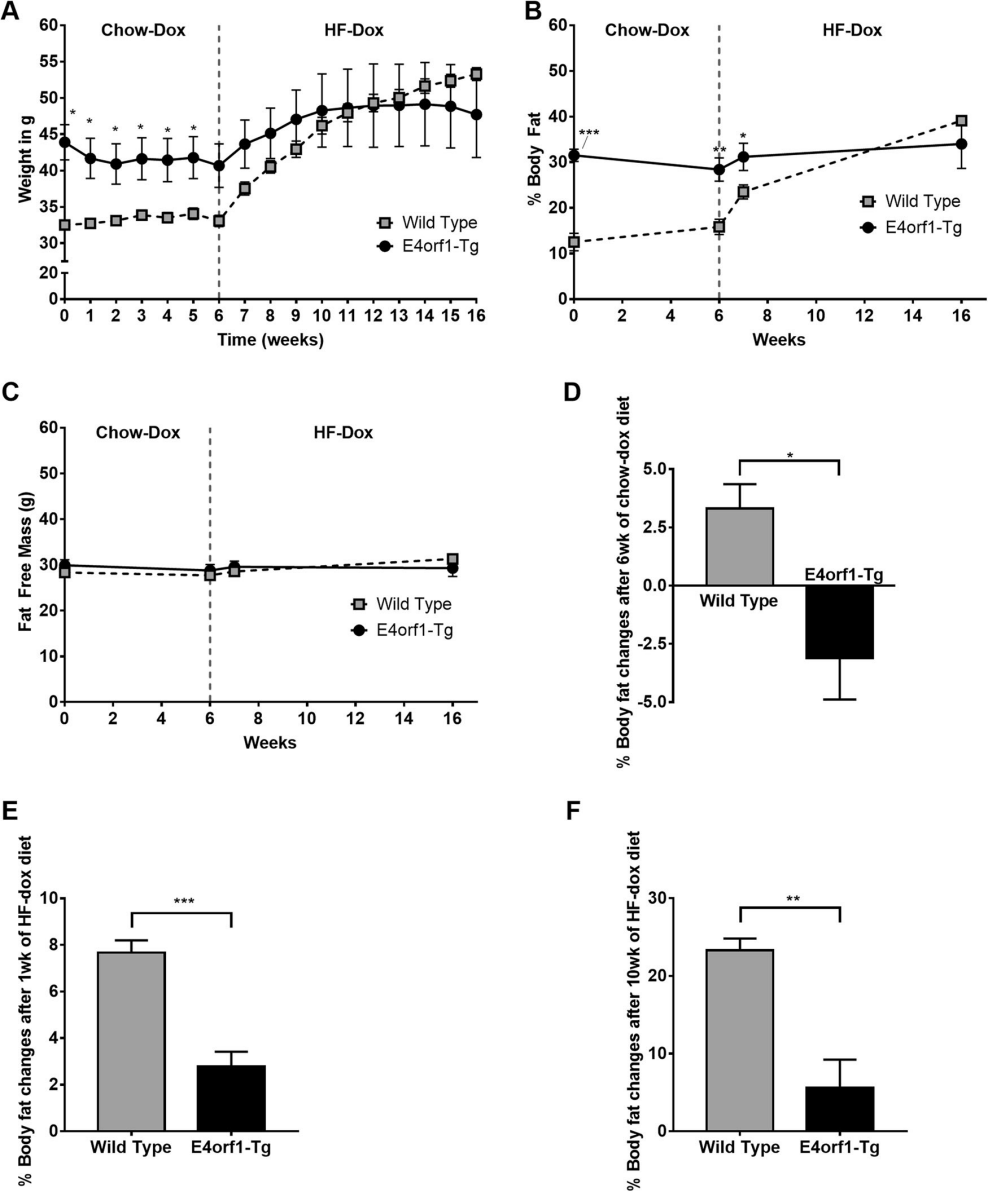
E4orf1-Tg mice show lower gain in body fat and body weight on high-fat diet. **a** Body weight changes of WT and E4orf1-Tg mice. **b** % Body fat changes of WT and E4orf1-Tg mice. **c** % fat-free mass changes of WT and E4orf1-Tg mice. **d** % Body fat changes after 6 weeks of chow-dox diet. **e** % Body fat changes after 1 week of HF-dox diet. **f** % Body fat changes after 10 weeks of HF-dox diet. Welch’s *t* test: *p < 0.05, **p < 0.01, ***p < 0.001.

At baseline, E4orf1-Tg mice were heavier compared to age-matched WT mice. This phenotypic difference might be attributed to the transgenic modification in E4orf1-Tg mice, breeding colony, or housing till recruitment in the study. Upon E4orf1 induction, E4orf1-Tg mice reduced body weight and % body fat and were protected against body weight and % body fat gain during HF feeding (Fig. 1a, b) compared to WT. After 6 weeks of chow-dox diet, E4 mice lost weight (−3.22 ± 0.96 vs. 0.55 ± 0.48 g, *p* < 0.01) and % body fat (Fig. 1d), while weight remain unchanged but % body fat increased in WT mice. The fat-free mass was unchanged in both the groups (Fig. 1c). Next, we tested the response of these mice to HF-dox diet. Following 1 week of HF-dox diet, gain in body weight (2.97 ± 0.75 vs. 4.46 ± 0.38 g, *p* = 0.12) and fat-free mass (0.80 ± 0.36 vs. 0.86 ± 0.19 g, *p* = 0.26) in E4orf1-Tg and WT mice, respectively, was not significantly different, but the increase in % body fat (Fig. 1e) was significantly higher in WT mice. During the 10 weeks, the HF-dox diet was continued, and compared with WT mice, E4orf1-Tg mice were protected against body weight gain (8.40 ± 3.75 vs. 20.22 ± 0.79 g, *p* < 0.05) and % body fat (Fig. 1f). We did not measure food intake or metabolic activity, but our previous study confirmed that there was no difference in these parameters for E4orf1-Tg mice^13^.

### Reduction in endogenous insulin requirement and improvement in glycemic control in normal chow or HF-fed conditions

**Fig. 2:**
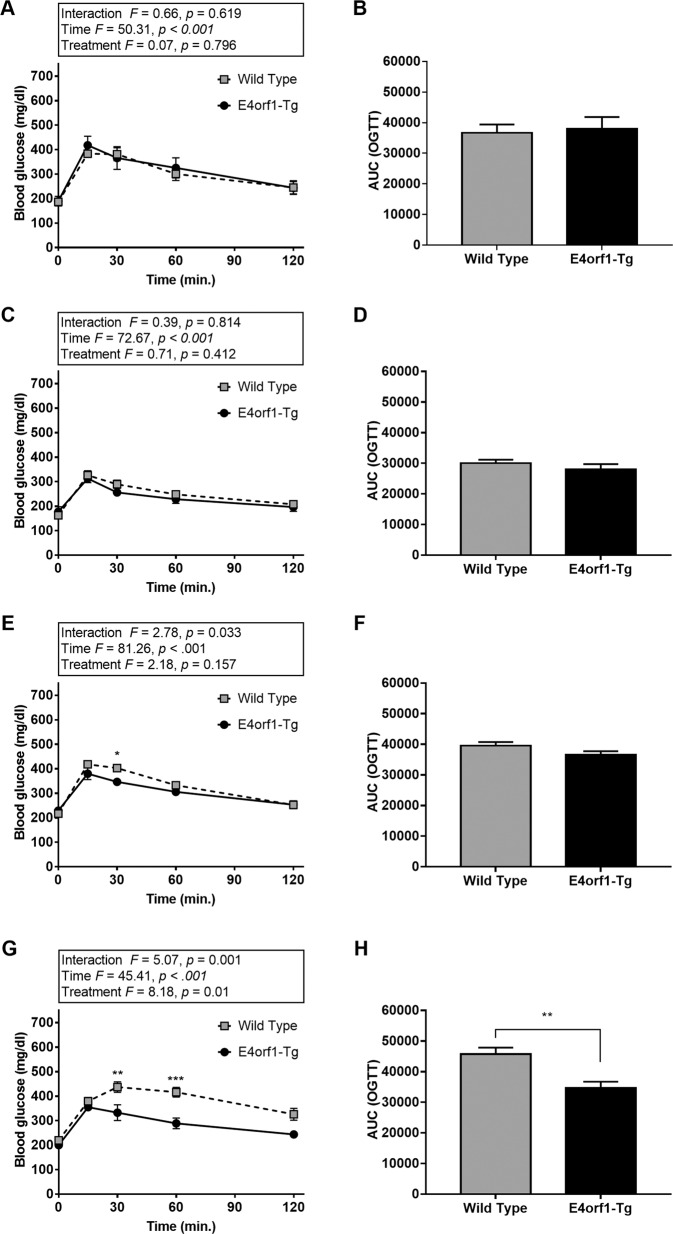
E4orf1-Tg mice improve glycemic control after high-fat diet feeding. Glucose tolerance tests and corresponding AUCs at **a**, **b** baseline. **c**, **d** After 6 weeks of chow-dox diet. **e**, **f** After 1 week of HF-dox diet. **g**, **h** After 10 weeks of HF-dox diet. Welch’s *t* test: *p < 0.05, **p < 0.01, ***p < 0.001.

**Fig. 3:**
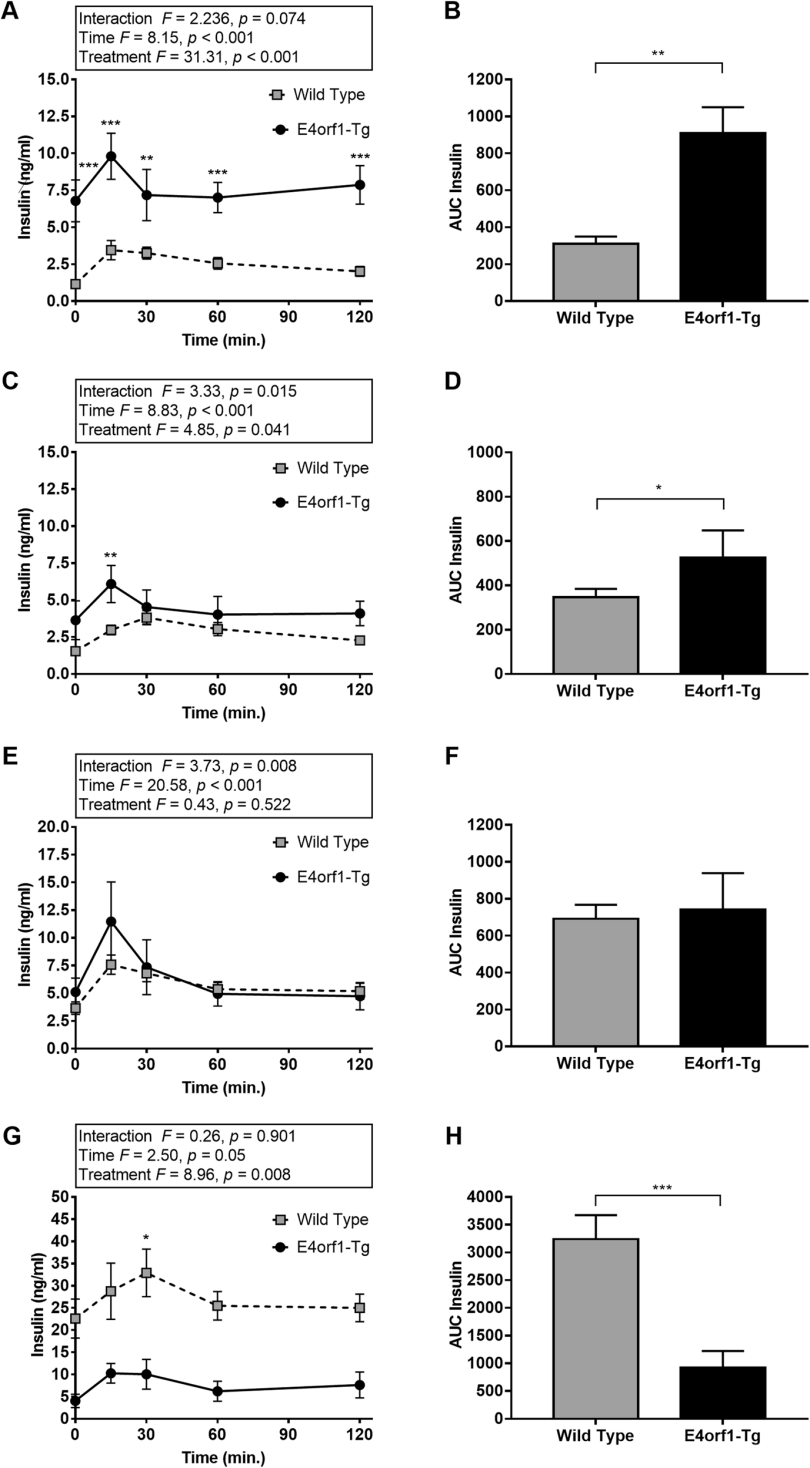
The requirement of endogenous insulin during GTT is reduced for E4orf1-Tg mice. Serum insulin level during GTTs and corresponding AUCs at **a**, **b** baseline. **c**, **d** After 6 weeks of chow-dox diet. **e**, **f** After 1 week of HF-dox diet. **g**, **h** After 10 weeks of HF-dox diet. Welch’s *t* test: *p < 0.05, **p < 0.01, ***p < 0.001.

Prior to induction of E4orf, baseline glucose clearance (Fig. 2a, b) did not differ between the two groups; however, the insulin requirement for glucose clearance in E4orf1-Tg mice was significantly higher (Fig. 3a, b) during baseline GTT compared with that in WT. The high insulin response in E4orf1-Tg mice may be due to relative insulin resistance resulted from higher body weight and % body fat in these mice. Following 6 weeks of E4orf1 activation with chow-dox diet, GTT showed no difference in glucose clearance (Fig. 2c, d) and insulin response (Fig. 3c, d) between the two groups. But the ΔAUC (changes in area under the characteristic curve (AUC) between GTTs) for glucose and insulin revealed lower requirement of insulin following a glucose load in E4orf1-Tg mice, which increased in WT mice (Supplementary Fig. 3b), while ΔAUC for glucose did not change (Supplementary Fig. 4a). Also, WT mice increased while E4orf1-Tg mice decreased HOMA-IR value (Supplementary Fig. 2a). These findings suggest that E4orf1 clears glucose and maintains lower secretion of insulin but does not induce hypoglycemia in normoglycemic mice.

**Fig. 4:**
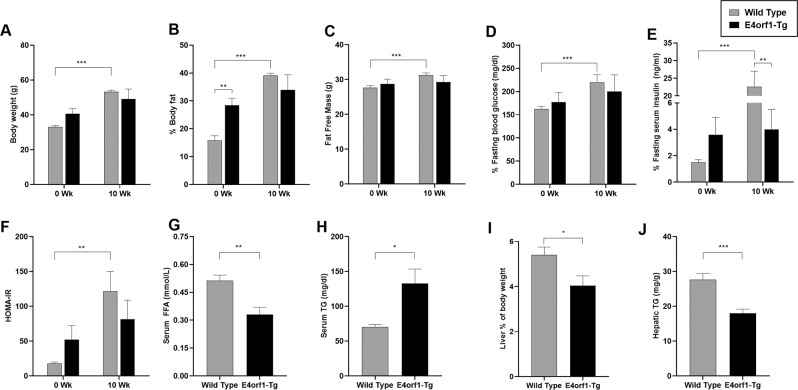
Body composition and markers of hepatic lipid metabolism after 10 weeks of HF-dox diet. **a** Body weight. **b** % Body fat changes. **c** Fat-free mass. **d** Fasting blood glucose level. **e** Fasting serum insulin level. **f** HOMA-IR. **g** Liver % of body weight. **h** FFA acid level in serum at termination. **i** Serum TG level at termination. **j** Hepatic TG level at termination. Welch’s *t* test: *p < 0.05, **p < 0.01, ***p < 0.001.

As expected, at 1 week after switching to HF-dox diet, glycemic control deteriorated in both groups as measured by ΔAUC for glucose and insulin (Supplementary Fig. 4c, d). No difference was observed in glucose clearance (Fig. 2e, f) or insulin levels (Fig. 3e, f) during GTT between the two groups. Glycemic control worsened after 10 weeks of HF-dox diet in both the groups; however, the deterioration for glucose and insulin AUC was significantly greater in WT compared to E4orf1-Tg mice (Supplementary Fig. 4e, f). E4orf1-Tg mice also demonstrated faster glucose clearance (Fig. 2g, h) and reduced insulin response (Fig. 3g, h) during the final GTT (16th week). Compared with E4orf1-Tg mice, WT increased fasting blood glucose level (Fig. 4d), fasting serum insulin (Fig. 4e), and HOMA-IR (Fig. 4f, Supplementary Fig. 2c) after the HF diet challenge.

### E4orf1 modulates lipid metabolism in the adipose tissue

As E4orf1-Tg mice lose % body fat during chow-dox feeding and show resistance to HF-dox-mediated increase in % body fat, we further tested lipid metabolism in the inguinal adipose tissue. Compared to WT mice, the mRNA expression of DNL-related enzymes—*Srebp1c*, *Fasn*, and *Acc1* (Supplementary Fig. 5a)—were lower in the E4orf1-Tg mice. Expression of lipolysis-related genes—*Atgl*, *Hsl*, and *Cgi-58*—was not different between the two groups (Supplementary Fig. 5b) except perilipin, which was significantly downregulated in E4orf1-Tg mice. Although *Atgl* mRNA expression was not different, its protein expression was significantly higher in E4orf1-Tg mice (Supplementary Fig. 5e). Among the genes indicating fat oxidation (supplementary Fig. 5c), the expression levels of *Ppar-γ*, *Ppar-α*, and *Cpt1a* were significantly downregulated in E4orf1-Tg mice suggesting reduced fat oxidation. Expression of genes related to inflammation including *Tnf-α*, *Il-6*, and *Mcp-1* was not different between the two groups of mice (Supplementary Fig. 5d).

Adipocyte morphology determined by H&E staining of adipose tissue sections (Supplementary Fig. 5f) showed no significant differences for adipocyte area, adipocyte diameter, adipocyte number, and frequency distribution of adipocyte diameter between the two groups (Supplementary Fig. 5g–j).

### E4orf1 improves serum metabolites and liver outcomes

E4orf1-Tg mice had lower serum FFA (Fig. 4g) and higher serum TG (Fig. 4h), even though they had lower liver weight (% of body weight) (Fig. 4i) and lower hepatic TG (Fig. 4j) compared to WT mice.

#### Liver histology

**Fig. 5:**
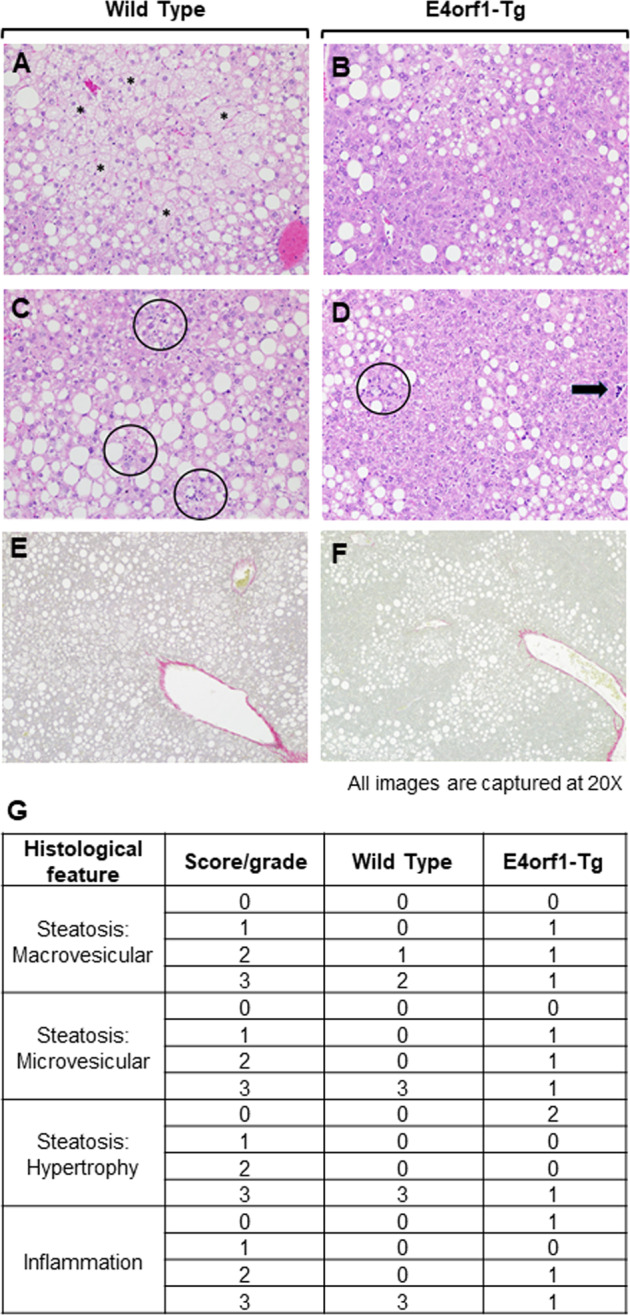
E4orf1-Tg mice have lower hepatic steatosis with possible protection from inflammation. First and second column are representative slides (×20) from WT and E4orf1-Tg mice, respectively. **a**, **b** Macrovesicular steatosis (large clear droplets) and microvesicular steatosis often with hypertrophy (asterisk (*)). **c**, **d** Presence of cellular infiltrates (inflammation—circled, hematopoiesis—arrow). **e**, **f** Fibrosis. **g** Scoring of liver histological features.

To determine hepatic steatosis, formalin-fixed liver sections were H&E stained and microscopic scoring was performed. Observed microscopic changes were graded and summary scores were calculated for lesions indicative of macrovesicular steatosis, microvesicular steatosis, hepatocyte hypertrophy, and inflammation. H&E sections confirmed greater lipid accumulation in WT compared with E4orf1-Tg mice, similar with hepatic TG quantification. H&E sections confirmed more lipid accumulation in WT compared with E4orf1-Tg mice, which matches with hepatic TG quantification (Fig. 4j). WT mice received the most extensive, score 3, for macrovesicular and microvesicular lipidosis compared to E4orf1-Tg mice (Fig. 5c, d). In addition, WT mice had more extensive hepatocellular hypertrophy (due to the microvesicular lipid accumulation filling/expanding the cell). Classic steatitis (inflammation associated with lipid droplets (LDs)) was observed in all three WT mice but in only one E4orf1-Tg mouse (Fig. 5e, f). Fibrosis was not observed in any mouse.

#### Markers of liver metabolism

**Fig. 6:**
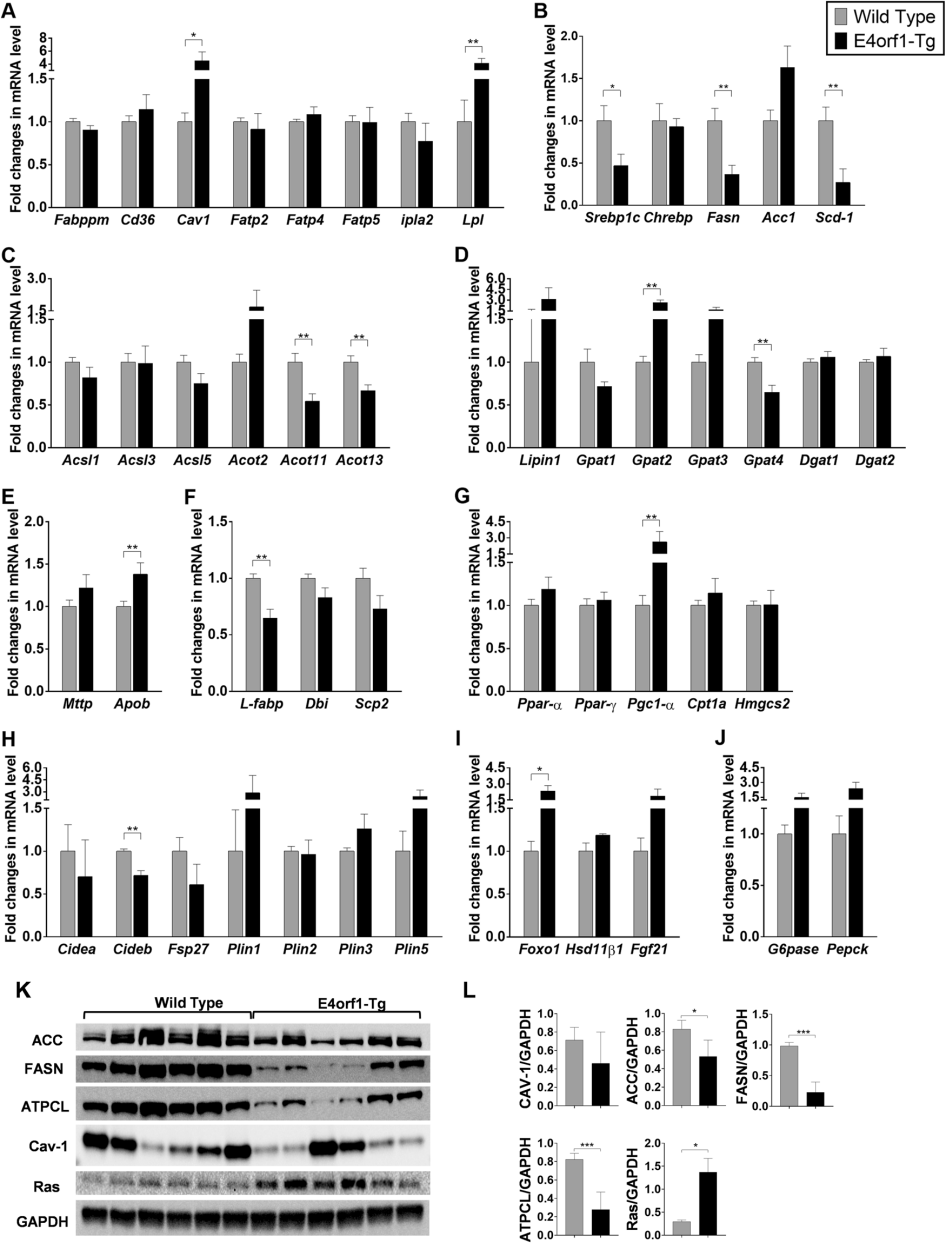
E4orf1-Tg mice modulate hepatic lipid metabolism favoring protection against hepatic steatosis. **a** Fatty acid uptake associate mRNA level. **b** DNL-associated mRNA expression. **c** Fat acid activation mRNA levels. **d** TG synthesis-related mRNA expression. **e** VLDL assembly- and secretion-associated mRNA levels. **f** Intracellular fat transport-associated mRNA levels. **g** Fat oxidation-associated mRNA expression. **h** Expression of mRNA associated with lipid droplet biology. **i** Expression of *Foxo1*, *Hsd11β1*, and *Fgf21* mRNA levels. **j** Gluconeogenesis-related mRNA level. **k**, **l** Levels of ACC, FASN, ATPCL, Cav-1, and RAS were analyzed by western blotting; results of quantification are shown after normalization to the value of GAPDH. Welch’s *t* test: *p < 0.05, **p < 0.01, ***p < 0.001.

As reported earlier^13–15^ in mice, E4orf1 protects against hepatic steatosis; however, the underlying mechanism is unclear. We compared the levels of selected hepatic protein (Fig. 6k, l) and mRNA associated with fatty acid uptake, DNL, fatty acid activation, intracellular fatty acid transport, TG synthesis, very low-density lipoprotein (VLDL) assembly and secretion, and LD formation in WT vs E4orf1-Tg mice, after 16 weeks of study. The results are as follows.

#### Fatty acid uptake

There was no significant difference in the mRNA expression of genes indicative of fatty acid uptake, namely, *Fabppm*, *Cd36*, *Fatp2*, *Fatp4*, *Fatp5*, and *ipla2*, except *Cav-1*, which was significantly upregulated in E4orf1-Tg mice (Fig. 6a).

#### De novo lipogenesis

The expression of *Srebp1c*, *Fasn*, and *Scd-1* was significantly lower in E4orf1-Tg mice, whereas no significant difference was detected for *Chrebp* and *Acc1* expression levels (Fig. 6b). Long-chain fatty acids are metabolized to acyl-CoA molecules involving an obligatory reaction known as thioesterification, which is catalyzed by various enzymes known as long-chain acyl-CoA synthetases (ACSLs). Expression of *Acsl* genes, specifically *Acsl1*, *Acsl3*, and *Acsl5*, were not different among the two groups (Fig. 6c). However, among acyl Co-A thioesterases (ACOTs), which are known for catalyzing the hydrolysis of acyl-CoA molecules into fatty acid and CoA, *Acot11* and *Acot13* genes were significantly downregulated in E4orf1-Tg mice compared to WT (Fig. 6c).

#### Intracellular lipid transport

Lipid-binding proteins are involved in the intracellular transport and partitioning of long-chain fatty acids and acyl-CoAs within hepatocytes. Among intracellular transport-associated genes, the mRNA expression of *L-fabp* was significantly lower in E4orf1-Tg mice compared to WT, while *Dbi* and *Scp-1* expression were not altered (Fig. 6f).

#### TG synthesis

The esterification process of fatty acids into glycerol-3-phosphate (G3P) is the major contributor of total TG synthesis in the liver. This esterification of long-chain acyl-coA into G3P is also considered as a rate-limiting step and catalyzed by G3P acyltransferase enzymes (GPATs). Among the *Gpats* (1–4), the expression of *Gpat2* was higher and *Gpat4* was lower in E4orf1-Tg mice compared to the WT (Fig. 6d), while there was no significant difference for *Gpat1* and *Gpat3*. Also, the mRNA level of *Dgat1* and *Dgat2*, which catalyze the acylation of diacylglycerol in the final step of TG synthesis, were not significantly different between the two groups (Fig. 6d). Another contributor of TG biosynthetic pathway, *Lipin-1* was elevated in E4orf1-Tg mice but did not reach statistical significance (Fig. 6d).

#### TG export

TG is exported from the liver via mature VLDL particles; briefly, TG is incorporated into *Apob* in the endoplasmic reticulum (ER) by *Mttp* followed by additional packaging of TG during the navigation from ER to Golgi apparatus. Interestingly, there was no significant difference between *Mttp* mRNA levels between the two groups, but *Apob* was significantly upregulated in E4orf1-Tg mice indicating increased lipid export from the liver (Fig. 6e).

#### Fat oxidation

Among the fat oxidation genes assessed, namely, *Ppar-α*, *Ppar-γ*, *Pgc1-α*, *Cpt1a*, and *Hmgcs2*, only *Pgc1-α* was significantly upregulated in E4orf1-Tg mice (Fig. 6g). LDs have the capacity to transiently store lipids in the liver, hence LD biology is very critical in overall lipid storage in the liver. Various LD-associated proteins are involved in the formation, expansion, and contraction among mRNA level of these proteins (perilipin and the DFF45-like effector/CIDE). No difference was found except for *Cideb*, which was significantly downregulated in E4orf1-Tg mice (Fig. 6h).

We also examined several genes implicated in NAFLD and/or associated with glucose and insulin metabolism such as *Foxo1*, *Hsd11β1*, *Fgf21*, *G6pase*, and *Pepck*; among these, only Foxo1 was upregulated in E4orf1-Tg mice (Fig. 6i).

## Discussion

This study confirms previous findings that E4orf1 improves glucose clearance in mice in the presence of HF diet and exhibits its insulin sparing action^13–16,18^. The activation of E4orf1 reduced insulin requirement during glucose load in chow-dox diet, which later continued in HF-dox diet, whereas WT mice exhibited continued increase throughout the study. Most importantly, it significantly advances our understanding about the crosstalk between adipose tissue and liver metabolism. It is noteworthy that the transgenic mice express E4orf1 specifically in adipose tissue and yet show improvement in hepatic steatosis^13^. A direct effect of E4orf1 on the liver is less likely in this experiment, as E4orf1 is neither secreted by adipose tissue nor taken up by cells and not present in the liver^17^. This is an example of how an intervention in adipose tissue may be used to indirectly influence hepatic lipid metabolism.

While the signaling crosstalk between adipose tissue and livers of these mice is not completely understood, one possibility is that the effect on liver is mediated via insulin. Generally, in vivo reduction in insulin concomitant with an improvement in glycemic control is due to the improvement in insulin sensitivity. However, E4orf1 upregulates cellular glucose uptake independent of proximal insulin signaling^18,19^ and does not improve insulin sensitivity^16–18^, yet it reduces endogenous insulin response^17^. The E4orf1-mediated reduction in insulin is not due to pancreatic beta cell damage or due to reduced ability of beta cells to secrete insulin^16,17^. Hence, E4orf1-expressing mice provide a model of improved glycemic control without an improvement in insulin sensitivity. This allows to study in isolation the possible effect of reduction in insulin amount on lipid metabolism in the liver. Considering the lipogenic properties of insulin, we hypothesized that the reduction in endogenous insulin response would reduce hepatic lipid storage.

Uptake of fatty acids received by the liver and their intracellular uptake; DNL; and synthesis, oxidation, and export of TG can collectively determine hepatic lipid stores. Liver histology showed that E4orf1 transgenic mice have reduced liver fat content compared to WT mice that also tend to have more pathological changes related to lipid accumulation. Although the changes at functional level are not confirmed, mRNA and protein signaling studies indicated the possibility that the reduction in hepatic lipid due to E4orf1 could involve reduced fatty acid uptake and intracellular lipid transport, greater TG export, and fatty acid oxidation. Greater circulating TG also support the possibility of TG export from the liver.

Our findings of reduced serum FFA level in E4orf1-Tg mice supports the reduced hepatic TG accumulation observed. At termination of the experiment, mRNA levels of lipolytic enzymes were not different between the two groups of mice, but the E4orf1-Tg mice showed increased ATGL protein expression in adipose tissue, suggesting increased lipolysis. Greater adipose tissue lipolysis is compatible with lower insulin response but not with reduced serum FFA in E4orf1-Tg mice. Increased *Cav-1* mRNA level in E4orf1-Tg mice liver suggests possible greater clearance of FFAs from serum. This may be balanced by greater lipid export from the liver, as suggested by greater *ApoB* mRNA in E4orf1-Tg mice, which suggests possible increased lipid turnover.

Increased lipogenesis is associated with the development of hepatic steatosis in humans and mice^24^. Thus lowering DNL is considered an effective mechanism to reduce hepatic steatosis. Here the collective downregulation (mRNA and/or protein) of Srebp1c, Acc, Fasn, Atpcl, and Scd-1 suggests reduced DNL in E4orf1-Tg mice, which might lead to significant reduction in liver TG.

We also found significant downregulation of *L-fabp* (also known as Fabp1) in E4orf1-Tg mice, which has anti-obesity, anti-hepatic steatosis, and anti-hyperglycemic effect in HF-fed mice^25^. In addition, we also reported upregulation of *Gpat2* and *Pgc1-α* and downregulation of *Acot11*, *Acot13*, *Gpat4*, and *Cideb* mRNA in E4orf1-Tg mice, which has protective effect against NAFLD development^26^.

Considering that hyperinsulinemia promotes fatty acid uptake and DNL and opposes TG export and fatty acid oxidation^27^, it is tempting to postulate that the effects of E4orf1 on hepatic lipid metabolism are mediated by lowering of endogenous insulin response. The causative directionality of this association cannot be established in this study. A study that determined the direct effect of E4orf1 transfection into HepG2 hepatocytes also indicated decreased DNL, greater fatty acid oxidation, and lipid export^28^. However, a direct effect of E4orf1 on the liver is less likely in the present experiment, as E4orf1 is neither secreted by adipose tissue nor taken up by cells^17^.

Conceptually, the study indicates that it is possible to influence hepatic lipid metabolism by altering adipose tissue metabolism. The effect appears to be mediated by reduced insulin response. Considering that inhibiting TG synthesis improves hepatic steatosis but increases liver injury^29^, it may instead be preferable to reduce hepatic steatosis by reducing associated hyperinsulinemia and that E4orf1 may be a candidate to induce such a change.

This study of substantial duration also provided new information about the role of E4orf1 in normoglycemia. Anti-diabetic agents such as insulin and sulfonylureas carry the risk of excessive hypoglycemia. Therefore, it would be important to determine whether a potential anti-diabetic agent such as E4orf1 can reduce hyperglycemia without reducing glucose below normal range. Previous studies of E4orf1-expressing mice showed significantly lower fasting glucose levels compared to that for the control group of mice^13^, but the levels for the transgenic mice were not in the hypoglycemic range. This study showed that E4orf1 reduces insulin response in mice that are hyperglycemic due to HF diet, as well as in normoglycemic mice on chow diet. However, the chow-fed mice remain normoglycemic. This is an important attribute of E4orf1 as a potential anti-diabetic agent.

In conclusion, the study supports our hypothesis that reduced endogenous insulin in E4orf1-Tg mice contributes to reduced hepatic steatosis. Collectively, this study underscores the possibility of indirectly impacting hepatic steatosis and identifies a strong candidate to achieve it.

## Supplementary information

Supplemental Material
