# Supplementary material for: Reducing endogenous insulin is linked with protection against hepatic steatosis in mice
Source: Nutr Diabetes. 2020 Apr 14;10:11. doi: 10.1038/s41387-020-0114-9 (PMC7156670; doi:10.1038/s41387-020-0114-9)
Supplement: Supplementary file 1 — Supplemental Material [file 41387_2020_114_MOESM1_ESM.docx]

**Supplementary Table 1: Primer sequences for genes used in RT-qPCR**

| **Gene name** |  | **Primer Sequence (5’-3’)** | **GenBank accession no.** |
| --- | --- | --- | --- |
| Fabppm | Forward | CGAGCAGTGGAAGGAGAT | NM_010325.3 |
|  | Reverse | GCAGAGGCAGACATTGATG |  |
| Cd36 | Forward | AGGTCTATCTACGCTGTGTTC | NM_001159555 |
|  | Reverse | AGGCATTGGCTGGAAGAA |  |
| Cav1 | Forward | AACCTCAACTGCCTACTCAA | NM_001243064.1 |
|  | Reverse | ACAGCAACCAATTCCTTAGATAAC |  |
| Fatp2 | Forward | AGGCGACATCTACTTCAACA | NM_011978.2 |
|  | Reverse | CCATACACATTCACTTCTTCAACA |  |
| Fatp4 | Forward | GACCAAGCCTACCTCACT | NM_011989.5 |
|  | Reverse | ACTGCCACATCTGCCATA |  |
| Fatp5 | Forward | AGCCAGCCATCTTATCACAT | NM_009512.2 |
|  | Reverse | AAGCAGCCAAGGAATCCA |  |
| Ipla2 | Forward | AACAGCACAGAGAATGAGGA | NM_001199023.1 |
|  | Reverse | GCACAGCGTAATGGAAGG |  |
| Lpl | Forward | CGCTCCATTCATCTCTTCATT | NM_008509.2 |
|  | Reverse | ACATCTTGCTGCTTCTCTTG |  |
| Srebp1c | Forward | GCTTCTCTTCTGCTTCTCTG | NM_001358314.1 |
|  | Reverse | GGCTGTAGGATGGTGAGT |  |
| Chrebp | Forward | TTCCACAAGCATCCTGACT | NM_001359237.1 |
|  | Reverse | AGAAGCGTGTTCACAAGTTG |  |
| Fasn | Forward | GTCGTCTATACCACTGCTTACT | NM_007988.3 |
|  | Reverse | ACACCACCTGAACCTGAG |  |
| Acc1 | Forward | GCAGCAGTTACACCACATAC | NM_133360.2 |
|  | Reverse | TCCGCCATCTTCCACAATA |  |
| Scd-1 | Forward | TGCCTCTTAGCCACTGAAT | NM_009127.4 |
|  | Reverse | ACTGTTGAGATGTGAGACTGT |  |
| Acsl1 | Forward | CAACACTGAAGGCGAAGAG | NM_001302163.1 |
|  | Reverse | CGAGGAGGATTGTGGAGAT |  |
| Acsl3 | Forward | AGGAAGATGTGTATATTGGCTACT | NM_001033606.2 |
|  | Reverse | CTGCTAATGTCTGTGGTGAAG |  |
| Acsl5 | Forward | CCATCTCCACTCCAGTCTT | NM_027976.2 |
|  | Reverse | TGTCAGCCACATCTTCCA |  |
| Acot2 | Forward | GATGGCTCTGGCTTATTACAAC | NM_134188.3 |
|  | Reverse | AGGTAGTTCACGGCTTCTT |  |
| Acot11 | Forward | CTGACTCTTGGCTCTACTTGT | NM_001347159.1 |
|  | Reverse | CTCTGAACCTCCGCTCTC |  |
| Acot13 | Forward | ACGAGAAGTAATGAAGGTTATGTT | NM_025790.2 |
|  | Reverse | AGATGCTGTCCACTAAGGT |  |
| Lipin1 | Forward | GCCGTGTCATATCAGCAAT | NM_001130412.1 |
|  | Reverse | ATCGCCAGAAGTAGAGGAG |  |
| Gpat1 | Forward | CTATCCAGTAACGAGTCCAGAA | NM_001356285.1 |
|  | Reverse | GGCGGTGAAGAGAATGTG |  |
| Gpat2 | Forward | GTCTTCCTACTGCTACTGTCA | NM_001081089.2 |
|  | Reverse | TGCTGTCTTCCTGTGTCA |  |
| Gpat3 | Forward | GCTTGCTGATTATAGGAACTACAC | NM_172715.3 |
|  | Reverse | GCTTGTTATGGTAATGGATGGT |  |
| Gpat4 | Forward | GGTGGAGAACAGCGAGTA | NM_018743.4 |
|  | Reverse | TCAGAAGGAAGGACAGAAGG |  |

**Supplementary Table 1: Primer sequences for genes used in RT-qPCR** (continued)

| **Gene name** |  | **Primer Sequence (5’-3’)** | **GenBank accession no.** |
| --- | --- | --- | --- |
| Dgat1 | Forward | GATTGGTGGAATGCTGAGTC | NM_010046.3 |
|  | Reverse | GGCTTGTAGAAGTGTCTGATG |  |
| Dgat2 | Forward | TCCAGAAGAAGTTCCAGAAGTAT | NM_026384.3 |
|  | Reverse | CAGGTGTCAGAGGAGAAGAG |  |
| Mttp | Forward | TCAAGGAGATGGCTGTTCA | NM_001163457.2 |
|  | Reverse | AGGATGTCAAGGCTGTATGT |  |
| Apob | Forward | TAGCAAGTTACAGAGCAGACA | NM_009693.2 |
|  | Reverse | AGTGACATCAACAGAGGAAGT |  |
| L-fabp | Forward | GTCAAGGCAGTCGTCAAG | NM_017399.5 |
|  | Reverse | ATGGTATTGGTGATTGTGTCTC |  |
| Dbi | Forward | CCTCAAGACTCAGCCAACT | NM_001037999.2 |
|  | Reverse | GTATTTACATCGCCCACAGTAG |  |
| Scp2 | Forward | GCCAGGAGATGCTATGAGA | NM_011327.4 |
|  | Reverse | CCAGTGCTTCGTAAGTGATG |  |
| Pppar-α | Forward | TCGCTATCCAGGCAGAAG | NM_001113418.1 |
|  | Reverse | ACAACAACAACAATAACCACAGA |  |
| Ppar-γ | Forward | CCACCAACTTCGGAATCAG | NM_001127330.2 |
|  | Reverse | GCTCTTGTGAATGGAATGTCT |  |
| Pgc-1α | Forward | ACAATAACAACAACAACCATACCA | NM_008904.2 |
|  | Reverse | ATTCTGTCTCTTGCCTCTTCA |  |
| Cpt1a | Forward | CAAGCCAGACGAAGAACATC | NM_013495.2 |
|  | Reverse | TGACCATAGCCATCCAGATT |  |
| Hmgcs2 | Forward | CTTGAACGAGTGGATGAGATG | NM_008256.4 |
|  | Reverse | CTATGAGGCTGCTGTGTCTA |  |
| Cidea | Forward | TCGGCTGTCTCAATGTCAA | NM_007702.2 |
|  | Reverse | GGATGGCTGCTCTTCTGTA |  |
| Cideb | Forward | GTGCTTGATGGTGCTTGA | NM_009894.3 |
|  | Reverse | CGATGTCCTTGCTGTGTT |  |
| Fsp27 | Forward | GCCACAGCACCAACTATG | NM_001301295.1 |
|  | Reverse | AATCTCCACCTCTAACACTCTC |  |
| Plin1 | Forward | GCGTGGAGAGTAAGGATGT | NM_175640.2 |
|  | Reverse | TGGTGCTGTTGTAGGTCTT |  |
| Plin3 | Forward | CGACAGGAGCAGAACTACT | NM_025836.3 |
|  | Reverse | CCGAGCACACTTGTTAGC |  |
| Plin5 | Forward | CACAGTGGAGGAGCAGAG | NM_001077348.1 |
|  | Reverse | AAGAGTGTTCATAGGCGAGAT |  |
| Foxo1 | Forward | GCTCTGTCCTGAAGAATCCT | NM_019739.3 |
|  | Reverse | CTAATCCTGCCACTGTCTGTA |  |
| Hsd11β1 | Forward | CCGTCATCTCCTCCTTGG | NM_001044751.1 |
|  | Reverse | CCTTGGTTATGTAGAGTTCTGTTC |  |
| Fgf21 | Forward | CCAAGACCAAGCAGGATTC | NM_020013.4 |
|  | Reverse | AGAGTCAGGACGCATAGC |  |
| G6pase | Forward | GGAAGGATGGAGGAAGGAAT | NM_008061.4 |
|  | Reverse | TCAGGTCAGCAATCACAGA |  |
| Pepck | Forward | GACATTGCCTGGATGAAGTT | NM_011044.3 |
|  | Reverse | CGTTGGTGAAGATGGTGTT |  |
| B2m | Forward | GAAGCCGAACATACTGAACTG | NM_009735.3 |
|  | Reverse | CTGAAGGACATATCTGACATCTCT |  |

**Supplementary Table 1: Primer sequences for genes used in RT-qPCR** (continued)

| **Gene name** |  | **Primer Sequence (5’-3’)** | **GenBank accession no.** |
| --- | --- | --- | --- |
| Tbp | Forward | CAACAACAGCAGGCAGTAG | [NM_013684.3](https://www.ncbi.nlm.nih.gov/nuccore/NM_013684.3) |
|  | Reverse | GGTGTGGCAGGAGTGATA |  |

**ABBREVIATIONS**

Fabppm, glutamatic-oxaloacetic transaminase 2, mitochondrial; Cd36, cluster of differentiation 36; Cav1, caveolin 1; Fatp2, solute carrier family 27 (fatty acid transporter), member 2; Fatp4, solute carrier family 27 (fatty acid transporter), member 4; Fatp5, solute carrier family 27 (fatty acid transporter), member 5; Ipla2, phospholipase A2, group VI; Lpl, lipoprotein lipase; Srebp1c, sterol regulatory element binding protein 1c; Chrebp, carbohydrate response element binding protein; Fasn, fatty acid synthase; Acc1, acyl CoA carboxylase 1; Scd1, stearoyl-Coenzyme A desaturase 1; Acsl1, acyl-CoA synthetase long-chain family member 1; Acsl3, acyl-CoA synthetase long-chain family member 3; Acsl5, acyl-CoA synthetase long-chain family member 5; Acot2, acyl-CoA thioesterase 2; Acot11, acyl-CoA thioesterase 11; Acot13, acyl-CoA thioesterase 13; Gpat1, glycerol-3-phosphate acyltransferase, mitochondrial; Gpat2, glycerol-3-phosphate acyltransferase 2, mitochondrial; Gpat3, glycerol-3-phosphate acyltransferase 3; Gpat4, glycerol-3-phosphate acyltransferase 4; Dgat1, diacylglycerol O-acyltransferase 1; Dgat2, diacylglycerol O-acyltransferase 2; Mttp, microsomal triglyceride transfer protein; Apob, apolipoprotein B; L-fabp, liver fatty acid binding protein; Dbi, diazepam binding inhibitor; Scp2, sterol carrier protein 2, liver; Ppar-α, Peroxisome proliferator-activated receptor alpha; Ppar-γ, peroxisome proliferator activated receptor gamma; Pgc-1α, peroxisome proliferative activated receptor, gamma, coactivator 1 alpha; Cpt1a, carnitine palmitoyltransferase Ia; Hmgcs2, 3-hydroxy-3-methylglutaryl-CoA synthase 2; Cidea, cell death-inducing DNA fragmentation factor, alpha subunit-like effector A; Cideb, cell death-inducing DNA fragmentation factor, alpha subunit-like effector B; Fsp27, cell death-inducing DFFA-like effector c; Plin1, perilipin 1; Plin3, perilipin 3; Plin5, perilipin 5; Foxo1, forkhead box O1; Hsd11b1, hydroxysteroid 11-beta dehydrogenase 1; Fgf21, fibroblast growth factor 21; G6pase, glucose 6-phosphatase; Pepck, phosphoenolpyruvate carboxykinase; B2m, Beta-2-Microglobulin; Tbp, TATA-box binding protein.

**Supplementary Figure 1:**


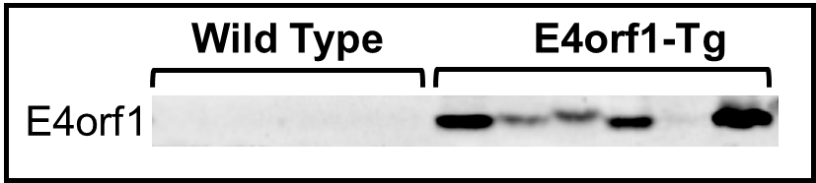


**Supplementary Fig. 1**: E4orf1 expression in the adipose tissue of E4orf1-Tg mice.

**Supplementary Figure 2:**





**Supplementary Fig. 2**: HOMA-IR changes in the study. (A) HOMA-IR value significantly decreased in the E4orf1-Tg mice after 6 weeks of chow-dox diet. **(B)** HOMA-IR value changes 1 week of HF diet. **(C)** The increase in HOMA-IR value of E4orf1-Tg mice was significantly lower after 10 week of HF diet. **(D)** Changes of HOMA-IR value throughout the study.

**Supplementary Figure 3:**

**
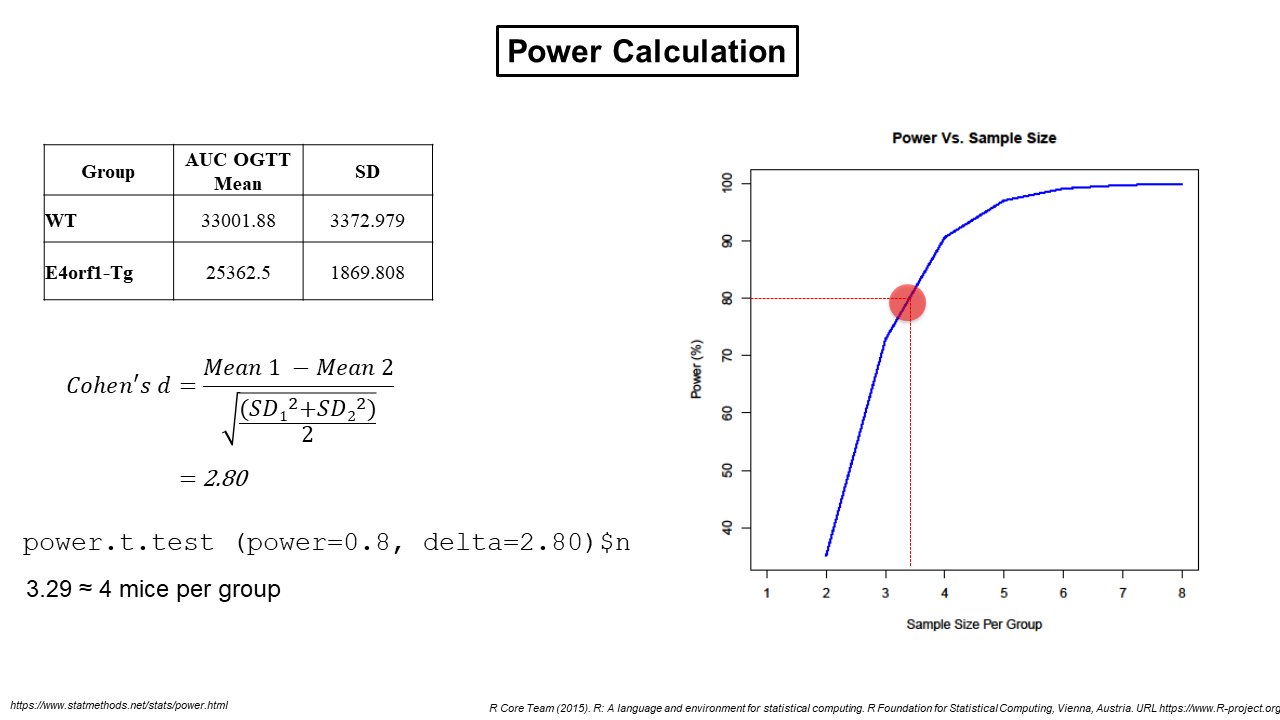
**

**Supplementary Figure 4:**

**

**

**Supplementary Fig. 4.** E4orf1-Tg mice improve glycemic control and reduces the endogenous insulin requirement. (A) Glucose AUC changes after 6 weeks of chow-dox diet. (B) Insulin AUC changes after 6 weeks of chow-dox diet. (C) Glucose AUC changes after 1 week of HF-dox diet. (D) Insulin AUC changes after 1 week of HF-dox diet. (E) Glucose AUC changes after 10 weeks of HF-dox diet. (F) Insulin AUC changes after 10 weeks of HF-dox diet. Welch’s *t* test: ***p*< 0.01, ****p*<0.001.

**Supplementary Figure 5:**

**
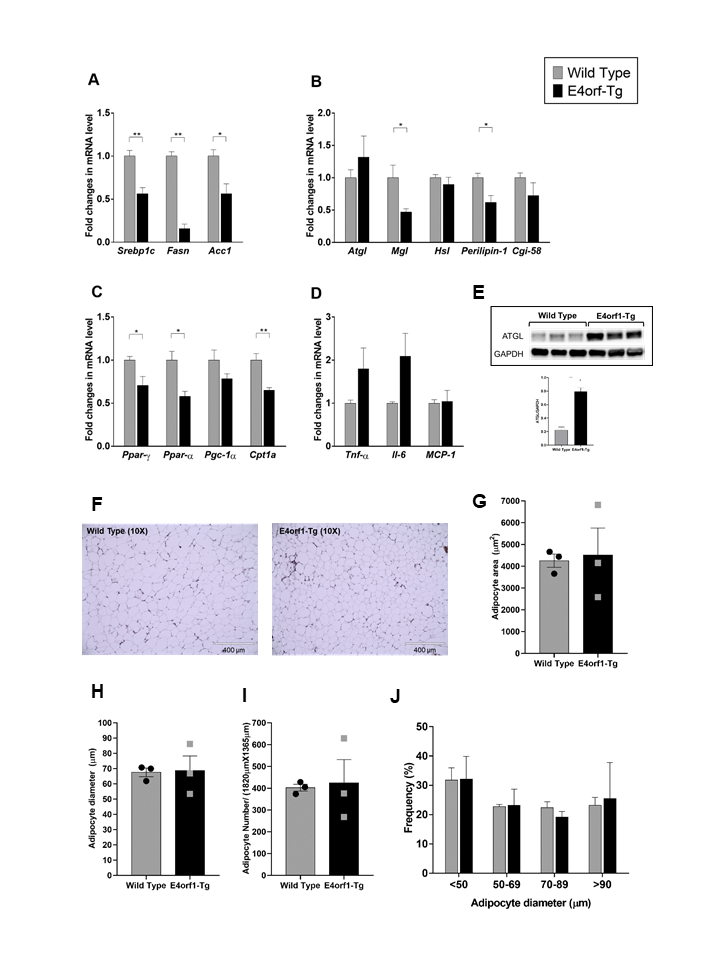
**

**Supplemental Fig. 5.** E4orf1-Tg mice increase markers of lipolysis and decrease de novo lipogenesis in adipose tissue. (A) *De novo* lipogenesis associated mRNA expression. (B) Lipolysis associated mRNA expression. (C) Fat oxidation related mRNA expression. (D) Inflammation associated mRNA expression. (E) ATGL protein expression. (F) H&E staining of adipose tissue, scale bar 400 μm. (G) Adipocyte area (μm^2^). (H) Adipocyte diameter (μm). (I) Adipocyte number. (J) Frequency of adipocyte diameter. Welch’s *t* test: **p*< 0.05, ***p*< 0.01, ****p*<0.001.
